# What aspects of periods are most bothersome for women reporting heavy menstrual bleeding? Community survey and qualitative study

**DOI:** 10.1186/1472-6874-7-8

**Published:** 2007-06-02

**Authors:** Miriam Santer, Sally Wyke, Pamela Warner

**Affiliations:** 1 MRC Health Services Research Fellow, Division of Community Health Sciences, University of Edinburgh Medical School, West Richmond Street, Edinburgh EH8 9DX, UK; 2 Director, Alliance for Self-Care Research, Department of Nursing and Midwifery, University of Stirling, Stirling FK9 4LA, UK; 3 Senior Lecturer in Medical Statistics, Division of Community Health Sciences, University of Edinburgh Medical School, Teviot Place, Edinburgh EH8 9AG, UK

## Abstract

**Background:**

Heavy menstrual bleeding is a common symptom amongst women of reproductive age, yet questions remain about why some women experience this as a problem while others do not. We investigated the concerns of women who reported heavy menstrual bleeding on questionnaire.

**Methods:**

A cross-sectional postal survey and qualitative interviews were carried out amongst a community-based sample of women in Lothian, Scotland. 906 women aged 25 to 44 reported heavy or very heavy periods in response to a postal survey of 2833 women registered with 19 general practices. Amongst those who had reported heavy menstrual bleeding, analysis was carried out of responses to the free text questionnaire item, "What bothers you most about your periods?" In addition, 32 of these women participated in qualitative interviews and their accounts were analysed to explore how menstrual symptoms and 'problems' with periods were experienced.

**Results:**

Even amongst this subgroup of women, selected on the basis of having reported their periods as heavy in the survey, pain was the aspect of their periods that 'most bothered' them, followed by heaviness, mood changes or tiredness, and irregularity or other issues of timing. Interviewees' accounts similarly suggested that a range of menstrual symptoms were problematic and some women did not disentangle which was worst. Judgements of periods as a problem were based on the impact of menstrual symptoms on daily life and this was contingent on social circumstances such as type of paid work and other responsibilities. Although women spoke readily of whether their periods were a problem, there was less clarity in accounts of whether or not menstrual loss was 'heavy'; women said they made judgements based on what was normal for them, degree of difficulty in containing blood loss and pattern of loss.

**Conclusion:**

Women with heavy periods are bothered by a range of menstrual symptoms and their impact on everyday life. Clinical emphasis should be on clarifying the presenting problem and providing help and advice for this, as well as on excluding serious disease. Sometimes simple approaches, such as help with analgesia, may be all that is required.

## Background

Heavy periods are an extremely common symptom, reported by 35% of women aged 25 to 44 [1]. Concerns have been raised about the number of women who receive surgical intervention for heavy menstrual bleeding, often in the absence of demonstrable pathology [2]. However, only approximately 6% of women consult regarding menstrual problems annually [3]. In the past it has been found that many women with troublesome menstrual symptoms did not consult their doctors [4], suggesting that some might be suffering unnecessarily for lack of treatment. More recently we have found that although 35% of women reported heavy periods, only 22% reported their periods as a marked or severe problem [1]. This raises the question why some women perceive heavy menstrual bleeding as a problem while others do not.

At present there is more evidence of a lack of understanding between patients with heavy menstrual bleeding and their doctors [5-7] than there is knowledge about their information needs and concerns. Qualitative research has shown dissatisfaction amongst consulters with the way their menstrual problems are assessed, in particular rejecting the medical emphasis on volume of loss in favour of impact of loss on everyday life [5]. Further, there might be additional menstrual symptoms. We found that overall 15% of our entire survey sample reported severe or very severe pain, whereas among the subgroup reporting heavy or very heavy periods this proportion was doubled (31%) [1]. A mismatch has been found between patients' and general practitioners' concerns about menstrual symptoms amongst women referred to secondary care; among cases where there was discordance between the woman and her general practitioner's reasons for clinic attendance, women were more likely to cite pain as a reason when her general practitioner did not and the general practitioner was very much more likely to cite heavy loss when the woman did not [7]. Research into heavy menstrual bleeding has generally focused on measured blood loss but it has been suggested that other outcomes might better reflect an improvement in health such as quality of life or patient satisfaction [8]. Others have found that this emphasis on assessing volume of menstrual loss extends into clinical practice [9].

It is important to understand the concerns of women with heavy menstrual bleeding as patient centred care has been shown to improve patients' enablement and satisfaction with health care [10] as well as health outcomes [11]. We aimed to investigate the concerns of women with heavy menstrual bleeding by carrying out a community-based questionnaire survey, plus qualitative interviews amongst a sample of women with self-reported heavy loss.

## Methods

A computer-generated random sample of 250 women aged 25 to 44 was selected from each of 19 general practice lists in Lothian, UK (or all women in target age range if fewer than 250). After exclusions, 4610 questionnaires were sent with one reminder. The data presented here are derived from questionnaire responses from the 906 women who reported their periods as 'heavy' or 'very heavy' on a fixed choice question which asked with respect to their periods over the previous six months; 'How heavy are your periods? (light loss; moderate loss; heavy loss; very heavy loss)'. Full details of this survey have been reported elsewhere [1].

In order to explore what constituted 'problem' periods among a large sample of women experiencing heavy menstrual bleeding, a free text question asked, 'What bothers you most about your periods?' This generated a wide variety of responses which were then coded. Coding schemes were developed by looking at 100 questionnaires and developing categories representing responses until these appeared to be saturated. Coding schemes were then checked with two other researchers for face validity. Categorisation involved difficult decisions; for instance, some had written 'can't be bothered with anything', or 'PMT' which could be interpreted as either mood changes or tiredness. These were therefore coded together as 'mood changes/tiredness'. Very few women wrote that the length of their periods bothered them most, so this was included with irregularity and other matters related to the timing of periods. A category including responses referring to 'accidents' could have been included with 'heavy', but some women had written 'worrying about accidents', which could perhaps refer to anxiety or social constraints rather than necessarily be associated with heaviness, so this was retained as a separate category.

In order to investigate how women spoke about judging their periods as problematic, qualitative interviews were conducted with 32 women who reported their periods as 'heavy' or 'very heavy' on questionnaire and had agreed to be contacted for interview. The qualitative sample was chosen such that all had reported their periods as heavy on a fixed choice questionnaire item and approximately half (14 of 32) had reported marked or severe period pain. Initial interviews were carried out amongst women who had or had not reported their periods as a problem but it transpired that more data was generated from those who had reported a problem. Later interviewees therefore focussed on this group such that 26 out of 32 of the final sample had reported periods as a problem. Overall, according to their questionnaire responses, 12 interviewees had consulted their general practitioner about periods within the previous 6 months. Of those who had not reported consulting on questionnaire, a further 12 said at interview that they had discussed periods with their general practitioner at some point in the past. Interviews lasted between 35 and 60 minutes and were carried out in women's homes. An interview guide was developed on the basis of the research questions, existing literature and findings from two preliminary focus groups covering: the impact, if any, of current menstrual symptoms; their history; how interviewees judged heaviness and 'problem'; self care and discussions with others, including consulting.

Interviewees were aged 27 to 45 and were white British except for one who was from Iraq. Twenty-five interviewees worked outside the home (six were nurses, five had clerical jobs, four retail, three factory, three academic and three other) and seven interviewees were not employed outside the home (three were caring for family, three were off work due to ill health and one described herself as unemployed).

Interviews were taped and transcribed before being entered into NVivo software. A coding frame was developed jointly by all three authors from relevant literature and themes arising in the first three interviews. The analysis used constant comparison to check for the presence of each theme in respondents' accounts and to seek counter examples [12]. The study was approved by the Lothian Research Ethics Committee. Names of interviewees have been changed to preserve anonymity.

## Results

In total, 2833/4610 women returned a completed questionnaire (response rate 61.5%), of whom 2574 reported having periods in the previous six months. Of these, 906 (35.2%) reported their periods as heavy or very heavy.

Table 1 shows the distribution of responses to the question 'What bothers you most about your periods?' There was a response rate of 89.6% to this question with 94/906 women either leaving it blank or writing 'nothing'. Even amongst women who had reported heavy or very heavy menstrual bleeding, pain remained the first concern for the greatest number with heaviness written by substantially fewer. Mood changes/tiredness were important to almost as many as heaviness. Overall, 44% (399) wrote in one thing that bothered them; 36.3% (329) wrote two things and 9.3% (84) wrote three or more things. Looking at whether an item was written in at all, rather than written as the first item, pain was written by 39.1% (354) and it appeared that mood changes/tiredness bothered marginally *more *women than heaviness (31.2% (282) compared with 29.0% (263)). When the subset of women who reported ***very ***heavy periods at survey was examined, this showed that for them, while heaviness was the aspect of periods most often identified as 'most bothersome', *fewer than half *did so (43%).

**Table 1:** Free text responses to question "What bothers you most about your periods?"

What bothers you most about your periods?* (First item written in)	**Women reporting heavy or very heavy menstrual bleeding**		**Women reporting very heavy menstrual bleeding**	**Women reporting heavy menstrual bleeding**
	
	**Frequency**	%	**% of 135**	**% of 771**
Pain	248	27.4	21	28
Heaviness	178	19.6	44	15
Mood changes/tiredness	158	17.4	10	19
Irregularity or other issues of timing	98	10.8	13	10
General inconvenience	52	5.7	2	6
Breast pain/swelling	25	2.8	2	3
Accidents	12	1.3	0	2
Other	41	4.5	2	5
Blank	94	10.4	5	11

**Total**	**906**		**135**	

*Examples of responses

Pain

e.g. "period pain", "stomach cramps", "backache" or similar

Mood changes/tiredness

e.g. "PMT", "irritability", "poor concentration", "tiredness", "unwell" or similar

Irregularity or other aspects of timing

e.g. "unpredictability", "duration", "spotting"

Heaviness

e.g. "heaviness", "amount of loss", "clots", "flooding"

General inconvenience

e.g. "interruption in usual activities", "having them", "hassle", "everything", "cost"

Accidents

e.g. "worrying about accidents", "leaking onto bedclothes"

Other

e.g. "pains in legs", "sickness", "diarrhoea", "headaches"

### Perception of menstrual 'problem'

When women with heavy periods were asked during qualitative interviews whether they felt their periods were a problem for them, they generally referred to the degree of impact that their periods had on everyday life. Inability to fulfil usual roles at home or in the workplace were central to discussions of periods as a problem. Joanne (table 2) was a self-employed single parent so felt work absence to be disastrous. Others spoke of the importance of flexibility at work to be able to change shifts, rest if necessary, make extra trips to the toilet or stay close to a toilet. Amongst interviewees who cared for young children, some obtained extra help during periods whereas others had more difficulty fulfilling their usual domestic roles. The overall impact of menstrual symptoms was linked to other social factors for some interviewees. For instance, Megan was concerned that lack of sleep due to pain if a period started at night would leave her unable to work the following day, whereas Patricia regularly slept on the (wipeable) sofa during her period in order to avoid staining her bed linen. However, she did not seem to view this as a particular problem as her daily activities were greatly restricted by chronic depression, for which she was on long term sick leave. Degree of impact on everyday life was therefore highly dependent on social context, such as employment status, type of work, extent of domestic responsibilities and level of support for these. Lifestyle factors were indeed also referred to by women who did not view periods as a problem, such as Sophie (table 2)

**Table 2:** Perception of menstrual 'problem'

*Miriam:* *And what is it that made you start to feel there was a problem, do you think?* *Joanne:* *Em, maybe having to take time off my work more than once or twice, or being more short-tempered with the kids as well*.

*Miriam:* *So do you think of them [periods] as a problem or an issue at all?* *Sophie:* *It doesn't really bother me that much now, because as it is, you know I've got my two little girls and I don't have an awful lot of time for things like sport and that sort of thing*.

*Miriam:* *Out of those things, the moodiness, the bloating, the heaviness and the pain that you get [all previously referred to by Anita], which of those do you actually find a bit of a problem?* *Anita:* *I would say the heaviness particularly when I am working is a problem. And also not so much the pain, the bloatedness in my stomach, I can manage that but the pains in my legs are very difficult when I'm working because I am on my feet for twelve and a half hour shifts and that is a long time when your legs are aching*.

It was not always clear from women's accounts exactly which aspect of menstrual experience was a problem for them. Interviewees often did not or could not distinguish between the impact of symptoms such as menstrual pain or general malaise and heavy menstrual bleeding (e.g. Anita, table 2).

### Judging periods as 'heavy'

Although women were generally quite clear about whether their periods were a problem or not, they expressed uncertainty regarding judgements of periods as heavy (see table 3). Women referred to volume of loss in discussing 'heaviness' but were uncertain what volume of loss was normal or what variations from their usual loss could be regarded as normal. Most women did not feel able to directly compare their blood loss with others, although many did attempt to estimate volume in terms of number and type of sanitary protection used and frequency of having to change this. Judging heaviness of loss was a complex decision based on personal norm (past experience of periods), difficulty in containing the volume of loss (sanitary protection used), and pattern of loss (reference was made by some women to clots and flooding).

**Table 3:** Judging periods as 'heavy'

*Miriam:* *And how heavy is it now?* *Katherine:* *Em, it's very difficult to quantify because it's not something you really discuss in lots of detail... I only know compared to what I usually have that well I suppose the major difference now is that I can't use just tampons, I have to have pads as well*...

*Miriam:* *And how is it that you know that they are heavy?* *Patricia:* *It's really hard to tell actually because you can't compare with anybody else. Em, but I know that if I use, I have to use the highest absorbency tampon and the highest absorbency pads and that still doesn't cope with it that well*.

*Miriam:* *And you said they were less heavy, in what way do you actually notice the difference?* *Sheila:* *Well there's not as big clots there, and at least I can stand and get washed and everything, before it was just running right down and I couldn't stand it, but I can get washed and that now, it's not as bad*.

## Discussion

Women reporting heavy menstrual bleeding wrote that they were as bothered by menstrual pain as they were by heavy loss. Qualitative interviews with women reporting heavy periods suggested that some found it difficult to distinguish which of their menstrual symptoms was the main cause of the impact of their periods on everyday life. Perception of periods as a problem was related to the impact of a range of symptoms on everyday life which, in turn, was linked to individuals' social circumstances such as type of employment or degree of flexibility around domestic responsibilities.

One strength of this study was its community setting which allowed us to explore concerns about menstrual symptoms amongst a population of women whose concerns were previously under-explored, most other studies having been carried out amongst those who had consulted or had been referred to secondary care. We acknowledge that our different sampling method might have yielded a more heterogeneous group of individuals than studies carried out amongst women recruited when they consult their general practitioners about menstrual symptoms [5] or amongst women attending secondary care [6,13]. One limitation of this sampling strategy is that it may have led to a greater focus on women with chronic rather than acute symptoms. In reflecting on the clinical implications of our findings, it is important to bear in mind the distinction between symptoms reported on questionnaire and symptoms which are reported in a consultation. The concerns of women in this study group may not necessarily mirror those of women with symptoms of more recent onset. Furthermore, although a response rate of 61.5% is about that which might be expected for a community survey on a topic which is not necessarily relevant to recipients, this does raise questions about non-responders. We have shown elsewhere [1] that non-responders were younger and likely to be living in an area of greater deprivation as calculated on the basis of postcode. They may have differed from responders in other ways also, with potential impact on our findings.

Free text questions can be criticised as being subject to interpretation by researchers. They are also more subject to response bias due to larger amounts of missing data than fixed choice questions. Women who responded to this question may differ from non-responders, for instance in motivation to complete the questionnaire, or in literacy. The large amount of missing data for this question is therefore very unlikely to have occurred randomly, limiting the generalisability of these findings. However, an open question inviting a free text response was a useful technique in allowing us to collect responses in women's own terms and without applying preconceived ideas. This study design enabled us to use the breadth of data from a large quantity of women to complement the greater depth of information from a smaller number of qualitative interviewees.

Among women consulting for menstrual problems the clinical focus on absolute volume of loss has been rejected in favour of a consideration of the overall impact of heaviness on everyday life elsewhere [5,13]. The findings presented here extend these findings to the wider community of women of reproductive age, showing the importance of assessing the impact on everyday life of a range of menstrual symptoms rather than heavy loss alone, even amongst women reporting heavy menstrual bleeding. The present findings are also consistent with our previously reported findings of a cumulative association of heaviness and pain on reporting periods a problem. That is, among 2123 women *not *reporting severe pain, but reporting heavy or *very *heavy periods, 21% and 72% respectively reported their periods a severe or marked problem [1]. Whereas, among 363 women reporting severe pain, and *also *reporting heavy or *very *heavy periods, corresponding percentages reporting periods a problem were 80% and 95% respectively [1].

## Conclusion

We found that the impact of a range of menstrual symptoms were important in understanding the concerns of women with heavy menstrual bleeding. Findings from studies of patient-centred consulting in other conditions show that a better understanding of the patient's illness experience contributes to improved patient satisfaction and outcomes [11]. This suggests that women would be helped by clinicians listening to women's accounts of their menstrual problems in their broadest sense, clarifying presenting symptoms and their impact on everyday life, and offering help and advice for these. Further research would help to explore different ways of delivering information to women to support self care and help them to manage menstrual problems themselves, as far as possible.

## Competing interests

The author(s) declare that they have no competing interests.

## Authors' contributions

All authors were responsible for the planning of the study, design of study tools and analysis and reporting of the study. MS conducted the qualitative interviews.

## Pre-publication history

The pre-publication history for this paper can be accessed here:



## References

[B1] Santer M, Warner P, Wyke S (2005). A Scottish postal survey suggested that the prevailing clinical preoccupation with heavy periods does not reflect the epidemiology of reported symptoms and problems. J Clin Epidemiology.

[B2] Coulter A, McPherson K, Vessey M (1988). Do British women undergo too many or too few hysterectomies?. Soc Sci Med.

[B3] Office of Population Censuses and Surveys Department of Health (1995). Morbidity statistics from general practice. Fourth national morbidity study 1991–1992. London, HMSO.

[B4] Scambler A, Scambler G (1985). Menstrual symptoms, attitudes and consulting behaviour. Social Science and Medicine.

[B5] O'Flynn N, Britten N (2000). Menorrhagia in general practice – disease or illness?. Soc Sci Med.

[B6] Protheroe J, Chew-Graham C (2005). The role of primary care in the diagnosis and management of menorrhagia: a qualitative study of women with menorrhagia. Primary Health Care Research and Development.

[B7] Warner P, Critchley HOD, Lumsden M-A, Campbell-Brown M, Douglas A, Murray G (2001). Referral for menstrual problems: cross sectional survey of symptoms, reasons for referral, and management. BMJ.

[B8] Lethaby A, Farquhar C (2003). Treatments for heavy menstrual bleeding. BMJ.

[B9] O'Flynn N, Britten N (2004). Diagnosing menstrual disorders: a qualitative study of the approach of primary care professionals. Br J Gen Practice.

[B10] Little P, Everitt H, Williamson I, Warner G, Moore M (2001). Observational study of effect of patient centredness and positive approach on outcomes of general practice consultations. BMJ.

[B11] Stewart M, Stewart M, Brown JB, Weston WW, et al (1995). Studies of health outcomes and patient-centred communication. Patient-centred medicine.

[B12] Seale C (1999). The quality of qualitative research.

[B13] Warner P, Critchley HOD, Lumsden M-A (2004). Is the 80 ml blood loss criterion useful in the management of complaint of menorrhagia?. Am J Obstetrics & Gynecology.

